# Regulation and dysregulation of immunoglobulin E: a molecular and clinical perspective

**DOI:** 10.1186/1476-7961-8-3

**Published:** 2010-02-23

**Authors:** Mariah B Pate, John Kelly Smith, David S Chi, Guha Krishnaswamy

**Affiliations:** 1 Division of Allergy and Immunology, Quillen College of Medicine, East Tennessee State University, Johnson City, TN 37614, USA; 2 Department of Medicine, Quillen College of Medicine, East Tennessee State University, Johnson City, TN 37614, USA; 3 James H. Quillen VA Medical Center, Mountain Home, TN, USA

## Abstract

**Background:**

Altered levels of Immunoglobulin E (IgE) represent a dysregulation of IgE synthesis and may be seen in a variety of immunological disorders. The object of this review is to summarize the historical and molecular aspects of IgE synthesis and the disorders associated with dysregulation of IgE production.

**Methods:**

Articles published in Medline/PubMed were searched with the keyword Immunoglobulin E and specific terms such as class switch recombination, deficiency and/or specific disease conditions (atopy, neoplasia, renal disease, myeloma, etc.). The selected papers included reviews, case reports, retrospective reviews and molecular mechanisms. Studies involving both sexes and all ages were included in the analysis.

**Results:**

Both very low and elevated levels of IgE may be seen in clinical practice. Major advancements have been made in our understanding of the molecular basis of IgE class switching including roles for T cells, cytokines and T regulatory (or Treg) cells in this process. Dysregulation of this process may result in either elevated IgE levels or IgE deficiency.

**Conclusion:**

Evaluation of a patient with elevated IgE must involve a detailed differential diagnosis and consideration of various immunological and non-immunological disorders. The use of appropriate tests will allow the correct diagnosis to be made. This can often assist in the development of tailored treatments.

## Introduction

**Figure 1:**
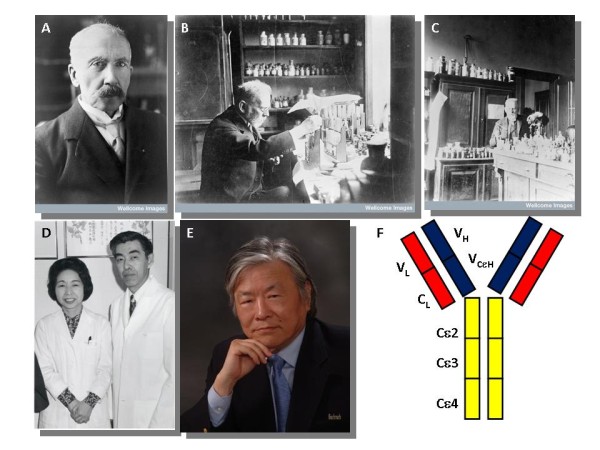
**Historical aspects of Immunoglobulin E**. Charles Richet (A-Credit: Wellcome Library, London: Charles Robert Richet), Paul Ehrlich (B and C-Wellcome Library, London Portrait of P. Ehrlich at work in his laboratory), Teruko and Kimishige Ishizaka (D- Courtesy of the Alan Mason Chesney Medical Archives, Johns Hopkins Medical Institutions), Susumu Tonegawa (E- Courtesy Dr. Susumu Tonegawa) and IgE molecule structure (F).

Immunoglobulin E has traditionally been associated with atopic disease and systemic anaphylaxis. However, its role in host defense, parasitic infection and immune surveillance suggest many other potential functions. The initial description of anaphylaxis was made by Portier and Richet in 1902 which led to Richet receiving the Nobel Prize for medicine in 1913 (Figure 1A). The mast cell was first described by Paul Ehrlich while experimenting with Aniline dyes as a medical student in 1878 (Figure 1B and 1C); he was awarded the Nobel Prize for his therapeutic discoveries in Medicine in 1908. The discovery of IgE by the Ishizakas (Figure 1D) in 1966 was a major advancement. Further understanding of IgE immunobiology was made possible by the description of class switch recombination (discussed later) by Susumu Tonegawa (Figure 1E), a Japanese scientist working in the United States. For this, he was awarded the Nobel Prize in Medicine in 1985.

## Molecular Regulation of IGE Production

**Figure 2:**
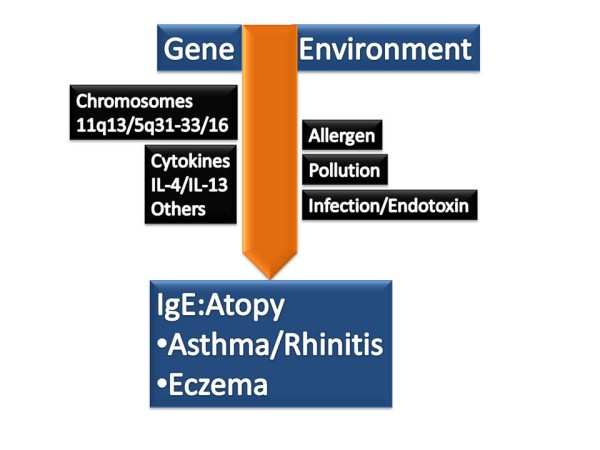
**Factors regulating IgE production**.

Immunoglobulin E is a class of immunoglobulin essential for the allergic response (Figure 1F). IgE is formed by the B lymphocyte and after several gene rearrangement steps is secreted. The production of IgE is regulated by genes, cytokines and the environment (Figure 2).

Immunoglobulin E consists of two identical heavy chains and two identical light chains with variable (V) and constant (C) regions (Figure 1F). The ε-heavy chains contain one variable heavy chain and four constant region domains (Cε 1-4). Immunoglobulin domains each contain around 110 amino acids and are beta sheets with three and four beta strands in the C type topology [1]. IgE is a component of a network of proteins involved in the signaling response to an allergen/antigen. These proteins include FcεRI, the high affinity receptor for IgE, CD23 (also known as FcεRII), the low affinity receptor for IgE, and galactin-3, the IgE and FcεRI binding protein. The known physiological properties of IgE are summarized in Table 1. Binding of IgE to FcεRI on mast cells and basophils induces signaling and leads to mast cell degranulation and mediator release. These include proteases, lipid mediators, and a plethora of cytokines, chemokines and growth factors. These mediators are partially responsible for eosinophil activation and survival seen in many disorders associated with elevated IgE [2-6].

**Table 1:** The Physiological Properties of Immunoglobulin E

General Characteristics	Molecular weight: 190,000 Da (170 kDa protein; 20 kDa Carbohydrate) Type: Monomer Subclasses: None
**Biology**	Does not fix complement Does not cross the placental barrier Half-life: 2 days Isoforms: Secreted and membrane bound IgE Structure: Two light chains (κ or λ) and 2 heavy chains (ε)

**Function**	Binds to High affinity IgE receptor (FcεRI) and degranulates mast cells and basophils Immediate Hypersensitivity IgE-mediated antigen presentation via FcεRI

### Cell-Cell Interactions in IgE Synthesis

**Figure 3:**
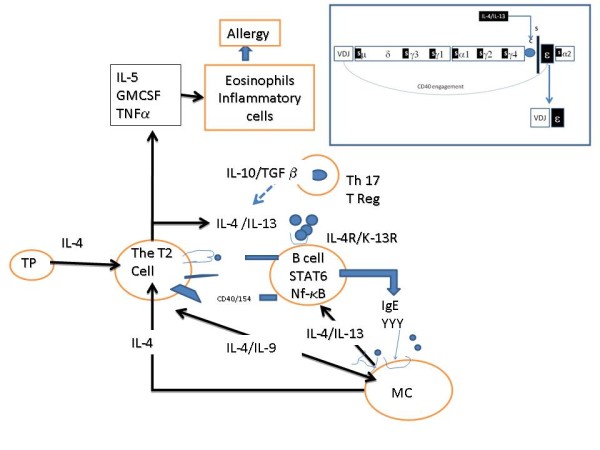
**T-B cell interactions, immune synapse (Prepared for the manuscript by Rahul Krishnaswamy) and IgE class switch recombination (shown in inset)**.

In the accepted model, an antigen/allergen is presented by a B cell, in the context of MHC class II molecules, to a Th2 cell, which recognizes the antigen via its T cell receptor (TcR)/CD3 complex. This leads to the expression of CD154 (or CD40 ligand) on the T cell, which engages the counter-receptor, CD40, to be expressed on B cells. This engagement of TcR/CD3, MHC II, antigen/peptide, CD154 and CD40 at the "immune synapse" leads to a sequence of events culminating in IgE secretion by the B cell (Figure 3). The sequential events include induction of CD 80/86 on the B cell that engages CD28 on the T cell, leading to transcription of pivotal Th2 cytokines IL-4 and/or IL-13. Following secretion, these cytokines bind to corresponding receptors (IL-13R or IL-4R) on the B cell, leading to STAT6 activation in B cells. This synergizes with Nf-κB, activated via switch receptors (CD40 and others), to induce activation-induced cytosine deaminase (AID) which induces class switch recombination (Figure 3) and activates germline transcription of Cε.

### IgE Class-Switch Recombination

A two-step process of DNA excision and ligation are required for assembly of a functional IgE. In the primary response, characterized by expression of membrane IgM and IgD, VDJ (heavy chain) and VJ (light chain) recombination occurs in fetal tissue (liver and bone marrow). This is both an antigen and a T cell-independent process. In the secondary immune response, which results in formation of the isotypes IgG, IgA and IgE, class switch recombination (CSR) occurs in secondary lymphoid tissues (lymphoid tissue, spleen and tonsils). This is T cell/cytokine dependent and an antigen dependent process. This results in high affinity antibodies, further modified by the process of somatic hypermutation (SHM). SHM results from missense mutations in the V regions of the immunoglobulin molecule.

First, during the pre-B cell stage, the individual heavy chain variable (V_H_), diversity (D) and joining (J_H_) exons randomly combined to form a V_H_(D)J_H _cassette that encodes an antigen-specific V_H _domain. This cassette is upstream of the constant μ exons and allows for the assembly of complete VDJ exons that encode an antigen-binding V_H _domain which produces intact μ heavy chains. The second step, class-switch recombination, is required for mature B cells to alter the isotope of their antibodies, while retaining their antigen specificity. This involves tightly regulated and irreversible exchange of the various isotope's VHJ cassette to construct different heavy chains [7]. The Cε locus of IgE is similar to other C_H _loci. The 5' region of each heavy chain isotope gene includes switch regions with tandem repeats, known as Sε and μ. In CSR, two switch regions, Sε and μ are combined, which allows the joining of the V_H_(D)J_H _and Cε regions. This joining generates a functional gene encoding IgE. CSR leading to IgE production is induced by cytokines IL-4 or IL-13 secreted by T helper 2 (TH2) cells [8].

### The Role of T cells, Cytokines and Tregs

**Figure 4:**
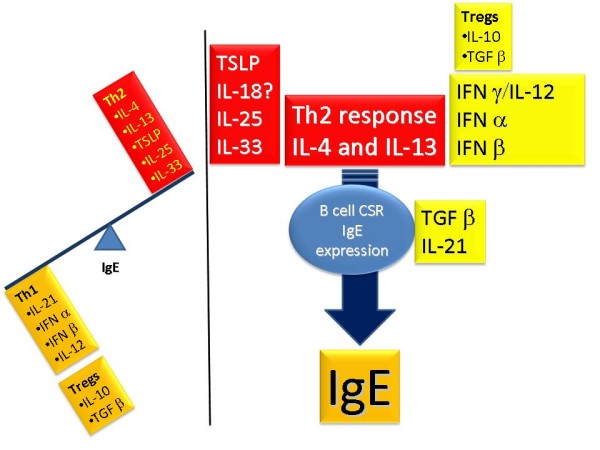
**Cytokine regulation of IgE production**.

Several T cell derived cytokines play a pivotal role in IgE CSR and expression (Figure 4). The cytokines that induce IgE CSR and/or IgE production in humans include: IL-4 and IL-13 (essential for CSR), TSLP (increases IL-4 and IL-13), IL-18 (increases IL-4 and IL-13 in some systems), IL-25 (increases IL-4 and IL-13) and IL-33 (increases IL-4 and IL-13). The authors showed that polymorphisms in chromosome 5q31.1 (Th2 cytokine cluster including IL-4 gene) were associated with IgE levels using sib-pair analysis [9]. The following cytokines inhibit IgE CSR and/or production: IFNγ, IL-10, IFN α and β (inhibit IgE production and also inhibit Th2 cytokine generation), TGF β and IL-21.

**Figure 5:**
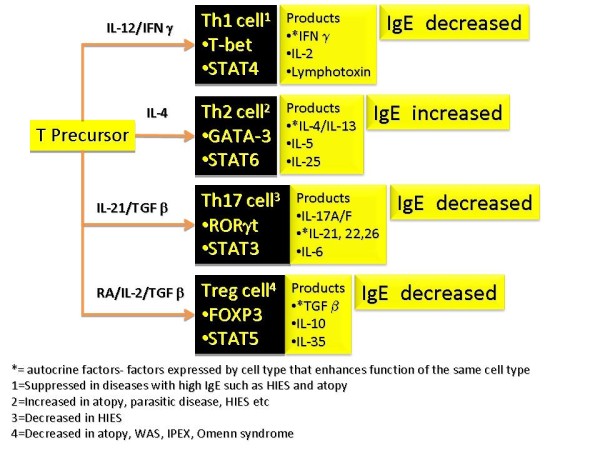
**T cell subsets that have effects on IgE (Refer to text for more details)**.

T regulatory (Treg) cells have important influences on the regulation of IgE synthesis. In the presence of specific growth factors and cytokines, T cell precursors can develop into Th1, Th2, Th17 and Treg cells (Figure 5). Th2 cells, regulated by GATA3 and STAT6 transcription factors, enhance IgE CSR (IL-4 and IL-13) and synthesis, while Th1 cells, regulated by T-bet and STAT4, inhibit the Th2-IgE axis. T cells with regulatory function include traditional Treg cells, Th3 cells (expressing TGF β) and Tr1 cells (peripherally-derived Treg cells expressing IL-10). These cells have negative regulatory effects on IgE synthesis. Tregs express CD25 and FOXP3 transcription factor and are thymically-derived. They develop from CD4+ precursor cells in the presence of retinoic acid (RA), TGF β and IL-2. By expressing TGFβ and IL-10, Tregs inhibit IgE CSR and synthesis.

## Ige Dysregulation

Normal levels of IgE are highly variable in the population. Factors regulating IgE levels include age, gene-by-environment interactions, genetic factors (such as certain polymorphisms), racial factors (higher levels are seen in African Americans and persons of Filipino descent), sex (males tending to have higher levels) and season (IgE levels may increase during pollen season in allergic individuals).

## Immune Dysregulation Associated with IGE Deficiency

IgE hypogammaglobulinemia is currently defined as a significant decrease in serum levels of IgE (<2.5 IU/mL) in a patient whose other immunoglobulin levels are normal (selective IgE deficiency) or diminished (mixed IgE deficiency). It is a laboratory finding that does not necessarily equate to a clinical disorder.

The prevalence of IgE deficiency is highly dependent on the population under study. The authors measured serum IgE levels in 500 Red Cross (RC) blood donors, 974 allergy-immunology (AIC) patients, and 155 rheumatology practice (RP) patients, and found that 2.6%, 8.1%, and 9.7% of these subjects, respectively, had undetectable levels of IgE. IgE deficiency was selective in 0.8% of RC donors, 3.1% of AIC patients, and 1.3% of RP patients, and mixed in 1.8%, 5.0% and 8.4% of these cohorts, respectively. Associated immunoglobulin deficiencies also varied with the population under study (Table 2). Low serum levels of IgE can also accompany other immunologic deficiency diseases, including common variable immunodeficiency, IgG subclass deficiencies, ataxia telangiectasia, and Bruton's hypogammaglobulinemia [10,11].

**Table 2:** Prevalence of IgE Hypogammaglobulinemia

	Selective deficiency	Mixed deficiency	Total	Common associated deficiencies*
AIC patients (N = 974)	3.1%	5.0%	8.1%	IgG4, IgG1, IgG2 & IgG3

RP patients (N = 155)	1.3%	8.4%	9.7%	IgA2, IgA1, IgG2, IgG4

RC donors (N = 500)	0.8%	1.8%	2.6%	IgG4

*In descending order of frequency

### Biological Significance

#### Prevention and control of infection

Several early reports suggested that isolated deficiencies in IgE predisposed to chronic sinopulmonary disease [12,13], whereas others found no such association [10,14]. At the time, there was no standard methodology in use for measuring IgE levels, nor did the authors of the reports use a common definition of what constitutes a true deficiency in this immunoglobulin. However, more recent reports using standardized technologies indicate that IgE antibody may play a protective role in some parasitic, bacterial, and viral infections in humans [15-19], and possess anti-tumor properties in vitro [20,21].

Secord and associates reported that the incidence of opportunistic infection and failure to thrive was lower in children with HIV-1 infection and high IgE levels than it was in HIV-1 infected children with low or normal IgE levels and similar decreases in CD4+ T lymphocyte counts; IgE anti-HIV antibody was detected in 43% of the children with high IgE levels[14]. Pellegrino and associates found that all members of a group of long-term pediatric survivors with maternally transmitted HIV infection had elevated total serum IgE levels and made anti-HIV-1 IgE capable of inhibiting HIV replication in vitro; the inhibitory effect was reversed when IgE was removed using immunoaffinity columns or anti-IgE antibody[15].

In a study involving 700 asymptomatic subjects from Tanzania, Bereczky and associates found that high IgE (but not IgG) anti-*P. falciparum *antibody was associated with a reduced risk for subsequent development of clinically evident malaria [16]. Duarte et al also found that *P*. *falciparum*-specific IgE responses contributed to the control of malaria, particularly in asymptomatic individuals [17]. There are also reports that IgE antibody can provide immunity against *B. burgdorferi *infection in children that lasts throughout adulthood [18], and contribute to the expulsion of intestinal parasites such as *N. americanus *[19]. The authors have found that IgE deficiency predisposes to sinopulmonary infection with common respiratory pathogens, including *Streptococcus pneumoniae*, *Haemophilus influenzae*, and *Moraxella catarrhalis *in patients of their allergy-immunology clinic [22].

#### Protection against autoimmune disease

**Figure 6:**
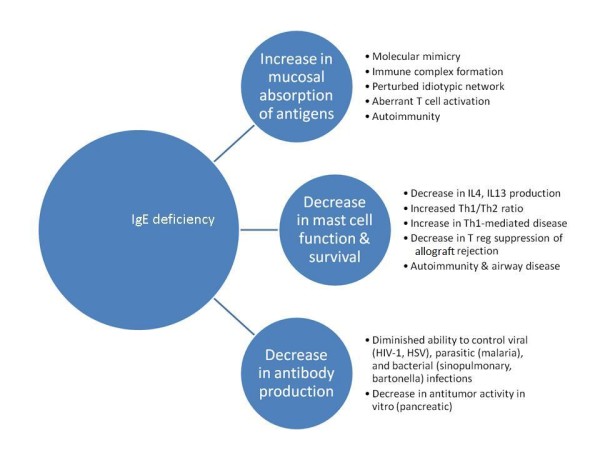
**Potential consequences of IgE hypogammaglobulinemia**.

The prevalence of autoimmune disease is recognized to be increased in persons with immunoglobulin deficiencies - particularly those with IgA hypogammaglobulinemia [23]. The authors have documented a similar predisposition in AIC patients with deficiencies in IgE [22]. There are potentially a number of mechanisms that could explain this association (Figure 6).

IgE is predominantly a mucosal immunoglobulin. Hence, as is postulated with IgA, it is possible that IgE protects against autoimmunization by preventing the systemic absorption of mucosal antigens [23]. A lack of antigen exclusion at the mucosal barrier could allow exogenous antigens to induce autoimmune responses by stimulating autoreactive lymphocytes through molecular mimicry [24,25]; by promoting immune complex formation [26]; by super-antigen-induced polyclonal activation of lymphocytes [27]; by inducing a perturbation of the idiotypic network [28]; and/or by aberrant induction of MHC class II antigens [29].

Evidence also indicates that rather than merely priming mast cells to respond to specific antigen, IgE, in the absence of cross-linking agents, favorably influences mast cell survival, receptor expression, and mediator release, and hence, has an important and active role in facilitating immune responses [30]. Mast cells have been shown to be essential intermediaries in Treg induced allograft tolerance in mice [31]; it is possible, therefore, that IgE deficiency predisposes to autoimmunity by adversely effecting mast cell survival and function. It is also possible that common genetic factors predispose an individual to both IgE deficiency and autoimmune disease, or that low levels of IgE merely reflect an imbalance between Th1 and Th2 lymphocyte activity. That, in turn, favors the development of Th1-mediated autoimmune diseases such as systemic lupus erythematosus and rheumatoid arthritis [32,33]. Systemic lupus erythematosus also may be related to Treg dysregulation, auto-antibody or anti-apoptotic defect.

#### Protection against reactive airway disease

The authors found that the prevalence of non-allergic reactive airway disease (rhinosinusitis, bronchitis and asthma) was increased in AIC patients with IgE deficiency. However, it was unclear as to whether these findings were the result of IgE deficiency or reflected selection bias inherent in allergy practices. In a study involving 664 pregnant women, Levin and associates found that the 21 individuals with low serum IgE (<2.0 IU/mL) had a higher prevalence of symptoms of rhinosinusitis, but a lower prevalence of physician diagnosed rhinosinusitis when compared to those with normal to elevated IgE levels [33]. Other studies on the prevalence of airway disease in IgE deficient patients are likewise inconclusive [10,12,34]. Experimental evidence is emerging that may provide an explanation for the occurrence of non-infectious, non-allergic airway inflammation in some IgE deficient patients. Kang and associates have demonstrated the occurrence of airway inflammation in lymphotoxin-deficient α (LTα-/-) mice, accompanied by diminished levels of IgE and reduced airway responsiveness, to both environmental and induced antigen challenge [35]. The lung inflammation in the LTα-/- mice is Th1-mediated and alleviated by reconstitution with IgE. Depletion of IgE in wild-type mice duplicates the lung pathologies of the LTα-/- mice, which is also reversed by the administration of IgE. The authors of this article suggest that the presence of low levels of IgE impairs the ability of mast cells to respond normally to airway antigens and, consequently, to produce cytokines that favor Th2 development (IL-4, IL-13); Th1 responses to the uncleared antigens then predominate.

### Clinical Features

In our experience, the majority of IgE deficient patients seek medical advice because of persistent sinorespiratory symptoms that are often assumed to be allergic in origin [22]. In our own Allergy Immunology clinic population, 79 IgE deficient patients have been identified. All of these patients tested negative on skin testing or in vitro allergy testing to a wide spectrum of indoor and outdoor allergens. When compared to a sex and aged-matched control group from the same clinic with normal levels of IgE, these subjects were more likely to complain of arthralgias, chronic fatigue, and symptoms suggestive of airway infection. In addition, they had a significantly higher prevalence of autoimmune disease and, as previously noted, non-allergic reactive airway disease. Sixty-two percent of the IgE deficient patients had depressed levels of other immunoglobulins, most commonly IgG4; 38 percent had selective IgE deficiencies. Not unexpectedly, serious infection involving both the upper and lower respiratory tract was more common in patients with low IgE and concomitant deficiencies in other immunoglobulins. Thus, in our experience, patients with IgE deficiency have a higher prevalence of sinopulmonary disease, chronic fatigue, arthralgias, autoimmune disease, and concomitant immunoglobulin deficiencies.

At the present time, attempts to replace IgE in persons with IgE hypogammaglobulinemia are neither feasible nor recommended. Rather, IgE deficient patients should be given standard therapy for their underlying conditions.

## Immune Dysregulation Associated with High IGE Levels

### Atopic Disease

Elevated levels of IgE may be seen in atopic disease, with the caveat that normal levels of IgE do not exclude atopy. Very high levels of IgE may be found in patients with food allergy, allergic fungal disease (such as sinobronchial airway mycoses or allergic fungal sinusitis) and atopic eczema. Table 3 lists conditions associated with elevated IgE levels, while Table 4 lists conditions with very high IgE levels and approaches to their evaluation.

**Table 3:** Elevated IgE: Etiologies and Evaluation

Main category	Sub-Category	Examples	Diagnosis
**Atopy**	Respiratory	Rhinitis, asthma, SAM	ST/RAST, PFT, Chest CT scan
Food allergy	Peanut/shrimp allergy	Food ST/RAST, Challenge	
Dermatological	Eczema, urticaria	RAST/Patch, biopsy, culture	
Other	Allergic Fungal Sinusitis	ST/RAST/Sinus imaging	
**Immune Deficiency**	Mixed T and B	Omenn syndrome	Flow, Immune tests
Syndromic	DiGeorge, WAS, HIES	Genetic, platelet, clinical	
Dysregulation	IPEX	Treg cell studies	
Humoral	Selective IgA deficiency	IgA level, functional antibody	
**Infection**	Bacterial	Pertussis, *S. Aureus*	Cultures, serology, clinical
Fungal	Aspergillus, Candida	Cultures, biopsy, serology	
Viral	EBV, CMV, HIV	Serology, PCR, cultures	
Mycobacteria	Leprosy, TB	Clinical, biopsy, culture	
**Parasitic infestation**	Helminth	Strongyloid, others	Clinical, serology, stool exam
Protozoan	Malaria	Clinical, blood smear	
**Malignancy**	Hematological	Myeloma, Lymphoma	SPEP***, Bone marrow
Solid tumor	Lung/colon/Breast	Radiology, biopsy	
**Inflammatory**	Vasculitides	Kawasaki, PAN*, CSS**	ANCA, biopsy
Inflammatory Arthritis	Rheumatoid arthritis	Rheumatoid factor, CCP****	
**Dermatological**	Blistering disease	Bullous pemphigoid	Biopsy, antibody
Idiopathic	Alopecia areata	Clinical, biopsy	
**Systemic disease**	Renal	Nephrotic syndrome	Urine protein, biopsy
Intoxication	Medications, alcohol	History, toxicology	
Pulmonary	Cystic fibrosis	CFTR Mutation, sweat chloride	
**Others**	Miscellaneous	RA, burns, Nicotine	Serology, history etc

* PAN- Polyarteritis nodasa, **CSS- Churg-Strauss Syndrome, ***SPEP- serum protein electrophoresis,

****CCP- cyclic citrullinated peptide

**Table 4:** Conditions with very high IgE levels

Extreme IgE Elevation	
Allergic fungal disease	Lympho-reticular Malignancy
HIV infection	Parasitic Disease
Atopic Dermatitis and Food Allergy	Netherton Syndrome
Hyper-IgE syndrome	IgE Myeloma

### Immune Deficiency Disease

Several immune deficiency disorders are associated with allergic manifestations. These include selective IgA deficiency and Common Variable Immunodeficiency. In addition, several primary immune deficiency disorders may demonstrate very high IgE levels[36]. These include Hyper-IgE syndromes (HIES), Immunodysregulation, polyendocrinopathy, enteropathy, X-linked syndrome (IPEX), The Wiskott-Aldrich Syndrome (WAS), Omenn syndrome and some forms of DiGeorge syndrome. **Hyper-IgE syndrome **[37-40] is characterized by highly elevated IgE levels, skin disease and repeated infections. IgE levels tend to exceed 10,000 U/mL, although a huge variability in levels may be observed. HIES syndrome can be idiopathic, autosomal dominant (AD) or autosomal recessive (AR). Most cases appear to have a sporadic basis, but mutations in the STAT3 gene is a feature of the autosomal dominant disorder (also referred to as type 1). AD HIES is characterized by typical skeletal changes such as "coarse facies", abnormal dentition and infection (Staphylococcal pneumonia and/or a pneumatocele). In AR HIES (also known as type 2), recurrent pneumonias, severe viral infections (Molluscum, Herpes simplex), neurological disease and vasculitis may be presenting features and mutations in the TYK2 gene may be seen.

**IPEX **is a rare syndrome mediated by a reduced or absent Treg population [36,41]. The syndrome manifests as early-onset enteritis (diarrhea), endocrinopathy (type 1 diabetes or hypothyroidism), elevated IgE levels and dermatitis/eczema. Hematological dyscrazias such as anemia, thrombocytopenia and eosinophilia are also observed. IPEX is secondary to mutations of the FOXP3 gene and a resultant deficiency of Treg cells. An increased Th2 response and elevated IgE levels are observed.

**Wiskott-Aldrich syndrome **is an X-linked disorder characterized by current infection, thrombocytopenia (with small platelets), neutropenia, eczema, high IgE levels, a very high prevalence of autoimmunity (including arthropathy, vasculitis, and inflammatory bowel disease) and malignancy. The defect lies in the WAS protein (or WASP) that is crucial to T cell, platelet and neutrophil function.

**Omenn syndrome **is a rare disorder presenting with recurrent infection, diarrhea, alopecia, eczema/erythroderma, lymphadenopathy, hepatosplenomegaly, eosinophilia and elevated IgE levels. Immune assessment shows elevated IgE levels in spite of deficiency in B cells numbers, panhypogammaglobulinemia, oligoclonal, nonfunctional T cell expansion and excessive Th2 skewing. The patients demonstrate one of several defects: mutations in RAG genes, Artemis gene, IL-7 receptor encoding gene and the RMRP gene (RNA component of mitochondrial RNA-processing endoribonuclease).

A subgroup of patients with **DiGeorge syndrome **may present not only with the profound T cell defect, seen with thymic aplasia, but also with findings consistent with Omenn syndrome (including elevated IgE levels and eosinophilia).

### Systemic Infections

Elevated IgE levels have been described in a variety of bacterial, fungal, mycobacterial and viral infections (listed in Table 4). Leprosy [42] and tuberculosis [43] have rarely been associated with elevated IgE levels [44,45]. Elevated IgE levels have also been described in viral infections (Epstein-Barr Virus and Cytomegalovirus). **HIV infection **is a well-recognized cause for elevated IgE levels [46-48]. Elevated IgE levels have been described in both adults and in children infected with HIV-1 [49], and are associated with a poorer prognosis [50]. A hyper-IgE-like syndrome and severe eczema have also been described with advanced HIV-1 infection [51].

### Parasitic Disease

Ascaris [52], Capillariasis [53], Paragonimiasis [54], Fasciola hepatica [55,56], Schistosomiasis [57,58], Hookworm (Trichuriasis) [59], Echinococcus [60], Onchocercariasis [61] and Malaria [62] have all been associated with elevated IgE levels. Of the many parasitic disorders, only a few are directly relevant to North American and these will be reviewed below. Giardiasis, Strongyloidiasis, *Trichinella spiralis *and Toxocara species occur with some frequency and have certain distinct and unique presentations.

**Strongyloidiasis **and its systemic consequences were reviewed by the authors recently [63]. Infection with *S. stercoralis *occurs when the skin of the feet contact free-living filariform larvae in the soil. The filariform larvae penetrate the skin and invade the blood vessels and subsequently enter the alveoli of the lung, where they are coughed up, swallowed and undergo maturation in the duodenum and jejunum. Over half the patients who harbor *S. stercoralis *have symptoms are related to the GI tract invasion, lung invasion or dissemination with strongyloid hyperinfection. The latter, usually seen in patients treated with glucocorticoids or immunosuppressive agents, can be fatal with complications such as sepsis, gram negative meningitis and/or respiratory distress [64,65]. Treatment with ivermectin (200 μg/Kg/day) is associated with a 90% cure rate.

**Toxocariasis **is a well recognized zoonotic disease mediated by the nematode belonging to the genus Toxocara. Adult worms are present in the intestinal tracts of dogs (*T. canis*) or cats (*T. cati*) and human infection is caused by egg ingestion [66,67]. Infective larvae migrate through the liver and lung and result in a plethora of allergic and inflammatory manifestations, referred to as visceral, ocular or cutaneous larva migrans. Eosinophilia, elevated IgE and involvement of brain, muscle, liver and lungs are responsible for the clinical manifestations. Treatment with albendazole or mebendazole and diethylcarbamazine may be attempted.

**Trichinellosis **is mediated by the nematode, *Trichinella spiralis*, transmitted by eating undercooked pork or larval forms present in cyst form in striated muscle [68]. Many patients may remain asymptomatic, while some patients develop abdominal pain, diarrhea, fever and excruciating myalgia (calf or masseter muscle). During the invasive stage of the illness, allergic phenomena such as urticaria or periorbital angioedema may occur. The disease is treated with albendazole and some studies have suggested a beneficial effect for glucocorticoids during the allergic and inflammatory stages of the disease.

***Giardia lamblia ***is a protozoan parasite that infects humans following the ingestion of infectious cysts (fecal-oral route or from contaminated food or well water). Symptoms include abdominal cramps, bloating, watery diarrhea and malabsorption. Elevated IgE levels and eosinophilia have been described [69]. Treatment with metronidazole, tinidazole, nitazoxanide or paramomycin may be variably effective, with paramomycin reserved for infected pregnant women.

### Neoplasia

A variety of neoplastic and hematological disorders have been associated with IgE.

**Solid tumors **such as cancers of the lung, colon, prostate and breast have been reported to elevate IgE levels [70]. These may be the result of dysregulation of the Th1/Th2-IgE axis [71]. Other neoplastic conditions known to present with elevated IgE levels include IgE myeloma and malignant lymphoma. Eosinophilia and elevated IL-4 and IgE levels have been shown in both Hodgkin's disease (serum IgE as well as intracellular IgE within Reed-Sternberg cells) and malignant/non-Hodgkin's lymphoma [72]. In multiple myeloma, polyclonal elevation of IgE is associated with improved survival [73].

**IgE myeloma **was first described in 1967 as an "atypical myeloma immunoglobulin" and since then several other cases of this rare myeloma have been reported [74]. The presentation of IgE myeloma is similar to that of an IgG myeloma, and most patients are diagnosed between the 6^th ^and 7^th ^decades of life.

### Inflammatory Disorders

Many other inflammatory disorders have been associated with elevated IgE levels. These include Kawasaki syndrome [75], vasculitides such as polyarteritis nodosa or Churg-Strauss syndrome[76], Guillian-Barre syndrome [77,78], inflammatory neuropathies [79], burns [80,81], Sjogren's syndrome [82], certain patients with rheumatoid arthritis who may also develop IgE rheumatoid factors [83], graft-versus-host disease and bone marrow transplantation [84] and scleroderma-like syndromes [85]. The prognostic or diagnostic role for IgE in these disorders is unknown

### Dermatological Disorders

Several dermatological and inflammatory disorders have been associated with IgE dysregulation and elevated IgE levels. Alopecia areata [86,87], erythema nodosum (especially due to streptococcus)[88], acral dermatitides and blistering diseases, such as pemphigus, have been associated with IgE elevation [89,90].

### Systemic and Toxic Disorders

Alcohol ingestion has been associated with elevated IgE levels [91]. Certain forms of nephritides demonstrate IgE elevation [92]. The elevation of IgE in these disorders may be due to dysregulation of the IL-4-IL-13 axis. In idiopathic nephritic syndrome, IL-13 levels are elevated, while in minimal change disease, polymorphisms of the IL-4 gene have been described [93]. Other conditions reported to show elevated IgE levels include cystic fibrosis [94] (with associated ABPA-like disease, probably secondary to increased airway penetration by allergen), nicotine abuse [95] and pulmonary hemosiderosis (Heiner's syndrome)[96].

## Conclusion

Major advancements have been made in our understanding of the molecular basis of IgE class switching including roles for T cells, cytokines and T regulatory cells in this process. Dysregulation of this process may result in either elevated IgE levels or IgE deficiency. Evaluation of a patient with elevated IgE must involve a detailed differential diagnosis and consideration of various immunological and non-immunological disorders. The use of appropriate tests will allow the correct diagnosis to be made. This can often assist in the development of tailored treatments.

## Competing interests

The authors declare that they have no competing interests.

## Authors' contributions

MP carried out some literature search, partially drafted the manuscript, and proofread the final version. JKS helped to draft the IgE deficiency aspect of the manuscript. DSC helped to draft the manuscript, revised it for important intellectual content, and assisted the finalizing of the manuscript. GK conceived and managed the study, drafted the manuscript, managed references, generated figures and tables, and has given final approval of the version to be published. All authors have read and approved the final manuscript.
